# Associating night-shift work with lifetime use of sleep medication and sleep quality in a cohort of female nurses

**DOI:** 10.1093/annweh/wxad058

**Published:** 2023-09-27

**Authors:** Daniëlla van de Langenberg, Jelle Vlaanderen, Nina Berentzen, Hans Kromhout, Roel Vermeulen

**Affiliations:** Exposure Science and the Exposome, IRAS, Institute for Risk Assessment Sciences, Utrecht University, Utrecht, the Netherlands; Exposure Science and the Exposome, IRAS, Institute for Risk Assessment Sciences, Utrecht University, Utrecht, the Netherlands; Flora van Leeuwen Epidemiology of Cancer, NKI, Netherlands Cancer Institute, Amsterdam, the Netherlands; Exposure Science and the Exposome, IRAS, Institute for Risk Assessment Sciences, Utrecht University, Utrecht, the Netherlands; Exposure Science and the Exposome, IRAS, Institute for Risk Assessment Sciences, Utrecht University, Utrecht, the Netherlands

**Keywords:** night work, occupational health, shift work sleep quality, sleep perception

## Abstract

Night-shift workers often sleep at moments, not in sync with their circadian rhythm. Though the acute effects of night-shift work on sleep quality directly after a night shift are well described, less is known about the chronic effects of night-shift work on sleep. We associated ever-working night shifts and recently working night shifts (<4 wk) with lifetime use of sleep medication and melatonin, self-reported average sleep duration and sleep quality over the 4 wk preceding inclusion (measured using the Medical Outcomes Study Sleep scale). We explored trends in sleep outcomes with average frequency of night shifts per week, tenure of night-shift works in years, and time since last performed night work. This research was conducted within the Nightingale study which is a Dutch cohort study of 59,947 female registered nurses aged 18 to 65. Working night shifts was not associated with self-reported nonoptimal sleep length and sleep quality. However, we observed higher odds of lifetime use of sleep medication for nurses who ever-worked night shifts (OR 1.24; 95% CI 1.13, 1.35) and who recently worked night shifts (OR 1.13; 95% CI 1.05, 1.22); with night-shift work frequency and tenure being associated with lifetime use of sleep medication (*P*-value for trend < 0.001 for both). Odds for melatonin use were elevated for nurses who ever worked night shifts (OR 1.55; 95% CI 1.40, 1.71) and recently worked night shifts (OR 1.72; 95% CI 1.59, 1,86). The findings of this study have practical implications for healthcare organizations that employ nurses working night shifts. The observed associations between night-shift work and increased lifetime use of prescribed sleep medication and melatonin highlight the need for targeted support and interventions to address the potential long-term sleep problems faced by these nurses.

**What’s Important About This Paper?**

This study of a large cohort of female registered nurses provides insight into the associations between night-shift work and sleep outcomes. The results demonstrate that while night-shift work does not directly affect sleep length and quality, it is associated with an increased likelihood of using sleep medication, indicating the potential long-term impact on sleep patterns. These findings have significant implications for promoting the health and well-being of shift workers, highlighting the importance of understanding and managing the effects of night-shift work on sleep.

## Introduction

Night-shift work has become an unavoidable part of our 24/7 society. In Europe 19% of the working population worked shifts including nights (defined as working for at least 2 h between 22:00 and 5:00) in 2019 (European Union Labour Force Survey—Access to Microdata—Eurostat 2023). The latest 6th EU Survey (6th European Working Conditions Survey—Publications Office of the EU 2023) observed the highest prevalence of shift work among healthcare workers (more than 40% of the workforce). Due to their working schedules, night workers need to sleep at moments, not in sync with their circadian cycle (Akerstedt 2003; Costa et al. 2010), which affects sleep mechanisms (Flo et al. 2013). Studies among night-shift workers have reported shorter sleep lengths (Pilcher et al. 2000), lower sleep quality (Åkerstedt and Wright 2009; Shao et al. 2010) and sleep disturbances (Korompeli et al. 2013; Ferri et al. 2016) directly after performing a night shift. Studies on the chronic effect of night-shift work on sleep are rare. Barak et al. associated a long history of night-shift work (>13.6 yr) with sleep disorders amongst female nurses (Barak et al. 2009). Former night-shift work has also been associated with reduced sleep quality (Dumont et al. 1997) and a disturbed sleep pattern (Ingre and Akerstedt 2004). Conversely, there is evidence that terminating shift work leads to less sleep disturbances (Linton et al. 2015) and the effects of night-shift work on fatigue might be reversible (Härmä et al*.*, 2018). Due to the lack of follow-up or longitudinal studies, not much is known about the reversibility of sleep disorders after night-shift work.

In this study, we assessed the long-term impact of night-shift work on sleep among female nurses by studying both self-reported sleep measures (i.e. duration and quality of sleep) and self-reported lifetime sleep medication use (melatonin and prescribed). Most existing studies on female nurses lacked the power to explore the impact of night-shift work on sleep mediation use (Uehli et al. 2014; Zhang et al. 2016). One large cohort study of Finnish public sector employees linked night-shift work with prescribed sleep medication, suggesting circadian disruption puts night workers at increased risk of developing clinically significant levels of sleep problems (Tucker et al. 2021). In our cohort study, we were able to study the effect of time since last performed night-shift work on sleep quality and duration. Our study also provided the opportunity to look at the effect of the frequency of night-shift work among current rotating night-shift workers. In addition, we assessed the potential for effect modification by age (Härmä et al. 2018; Tucker et al. 2021) and diurnal preference (e.g. morningness or eveningness) (Arendt 2010; Juda et al. 2013).

## Materials and Methods

### Study design

The Nightingale study is a large prospective cohort study of women holding a nursing certification. The rationale, study design, and baseline characteristics are described elsewhere (Pijpe et al. 2014). In brief, in 2011 all registered female nurses (national registry for healthcare professionals, BIG-register) in the Netherlands between the ages of 18 and 65 were invited to take part in this study, resulting in 192,931 eligible participants. The percentage of invitations that resulted in a response was 40% (*n* = 79,932). A response could be a decline, incomplete participation (i.e. informed consent yet less than half of the questionnaire completed), or complete participation (i.e. informed consent and at least half of the questionnaire completed including the section on occupational history and exposures and main confounding factors). Here we use the information at baseline from participants with “complete participation” (*n* = 59,947), who completed the web-based questionnaire between 6 October 2011 and 1 February 2012. The Nightingale study has been approved by the Medical Ethical Committee of the Netherlands Cancer Institute (Pijpe et al. 2014).

### Study variables

*Assessment of night-shift work*: We defined “ever working night shifts” as having worked at least 1 night shift per month for at least 6 mo during a lifetime. We defined a night shift as having worked at least 1 h between midnight and 6 AM. Job histories were used to determine the total number of years a participant has worked night shifts. We defined “currently working night shifts” as having performed night shifts during the past 6 mo. “Recently working in night shifts” was based on a question about the frequency of night shifts during the 4 wk preceding filling out the questionnaire. Possible answers ranged from “always” to “never” using a 6-item Likert scale. A dichotomous variable was created where the answers “never” and “seldom” were transformed into “no”, and the other categories (“sometimes”, “often”, “most of the time”, and “always”) were transformed into “yes”.

Tenure of night-shift work was analyzed per 5-yr increment (<5 yr (reference), 5–10 yr, 10–15 yr, 15–20 yr, and >20 yr). Duration in years since last performed night shifts was categorized into: <1 yr, 1–3 yr, 3–5 yr, 5–10 yr, 10–20 yr, and >20 yr. The frequency of night shifts was defined as how often the participants performed night shifts in the preceding 4 wk, among current night-shift workers. Six answer categories were available, including “never”, “seldom”, “sometimes”, “often”, “most of the time”, and “always”.

*Assessment of sleep outcomes*: We assessed the quality of sleep using different outcomes including sleep medication use, self-reported sleep quality using the Medical Outcomes Study (MOS) Sleep scale, and nonoptimal sleep length. We had dichotomous (yes/no) information on lifetime use of prescription sleep medication (specified as zopiclone, temazepam, oxazepam, lorazepam, or zolpidem) and sleep medication use during the period nurses worked night shifts. We created a specific period for participants when they were engaged in night shift work, and we related this period to their medication use during the same timeframe. We also had dichotomous (yes/no) information on the lifetime use of melatonin. Furthermore, melatonin period-specific information was reported: start- and stopping-ages and whether the use was daily or only during periods of circadian disruption.

We used the MOS Sleep scale to assess sleep quality over a period of 4 wk before filling out the questionnaire. The combination of 9 of the 12 MOS Sleep scale items converted to a 0 to 100 scale resulted in an overall sleep problem summary (SupplementaryS1 Appendix), previously defined as the sleep problems index II (SPI II) (Allen et al. 2009). A high SPI II score represents a troubled, difficult sleep. A score of > 25 on the SPI II was considered an indication of sleeping problems and was therefore used as a cutoff value (Smith and Wegener 2003; Hays et al. 2005; Kline et al. 2012). As part of the MOS Sleep scale, information on self-reported sleep length in hours on average during the past 4 wk was available (quantity of sleep, item 2, SupplementaryS1 Appendix). We defined less than 7 h or 9 h or more as nonoptimal sleep length (Viala-Danten et al. 2008a), since not only shorter but also longer sleep can be considered nonoptimal (Viala-Danten et al. 2008b). 4.6% of the participants of our study reported ≥9 h of sleep. Information on self-reported sleep length in hours between two consecutive night shifts was also available.

*Assessment of covariates*: A low level of education was defined as having an intermediate vocational education (community college), a medium level of education was defined as having a degree in nursing care in applied sciences and a high level of education was defined as having an academic degree (Pijpe et al. 2014). We used the highest achieved level of education. We asked the nurses to report their marital status and birth origin (place of birth). BMI was calculated using self-reported current height (cm) and body weight (kg). Physical activity included the number of hours the nurses reported to exercise per week, including walking and riding a bike. We assessed smoking duration in years and alcohol consumption by number of days per week participants consumed alcoholic beverages in the past year. Diurnal preference was self-assessed using an item of the Horne–Ostberg scale (Horne and Ostberg 1976) with 6 categories (including the answer categories “obvious morning preference”, “more morning than evening preference”, “more evening than morning preference”, “obvious evening preference”, “no specific type”, and “I don’t know what type I am”). We used a converted variable, combining the answer categories for morning preference together, combining the answer categories for evening preference together and combining “no specific type” with “I don’t know what type I am”. This self-assessment of one’s chronotype gave a similar result (*R*-square of 88, 0.8) to the 19-item questionnaire in a validation study (Roenneberg et al. 2007).

### Data preparation and analyses

*Missing data*: The number of missing observations varied between 0 and 8231 (13%) for the exposure variables assessing night work and the earlier described covariates. For the sleep outcome variables, a maximum of 9% of the variables were missing. We conducted multiple imputations using the R software package “multivariate imputation by chained equations” (MICE) with 5 iterations (Buuren and Groothuis-Oudshoorn 2011). We repeated this process of data imputation 5× and pooled the results. Furthermore, we analyzed the missing data in the outcome variables, looking for patterns indicating systematic missing data or selective dropout. However, we found no notable patterns, suggesting that the missing data were random and unrelated to participant characteristics or outcomes.

*Association between night work and sleep outcomes*: We analyzed dichotomous outcome variables (lifetime use of sleep medication, lifetime use of melatonin, nonoptimal sleep, and SPI II score > 25) using logistic regression analyses (GLM function of the LMER package for R (Bates et al. 2009) and calculated OR with 95% CI). All models were adjusted for BMI and age. In a second confounder model, analyses were also adjusted for a set of a priori determined confounding factors based on previous literature (Akerstedt 2003; Costaet al. 2010) including age, BMI, alcohol consumption, physical activity, marital status, birth origin (place of birth), highest achieved level of education, diurnal preference, and smoking. We investigated whether the impact of night work on sleep varied depending on age and diurnal preference (chronotype). To explore this, we predetermined these variables as important factors and tested them for effect modification. We stratified the data based on age and diurnal preference to separately analyze the relationship between night work and sleep within each subgroup. A *P*-value below 0.05 for the interaction term indicated statistically significant effect modification, suggesting that the association between night work and sleep outcomes differed based on age or diurnal preference. This approach allowed us to examine how age and chronotype influenced the relationship between night work and sleep outcomes. We explored whether the impact of night work on sleep was modified by age and diurnal preference (chronotype), by separately including an interaction term in the model and by stratifying the analyses for these potential modifiers. In addition, we performed logistic regression analyses to examine the association between night-shift work and sleep medication use. Specifically, we compared nurses who initiated sleep medication use during a period when they worked night shifts (including those who began using sleep medication for the first time during this period) with nurses who never used prescribed sleep medication. Additionally, we conducted analyses of sleep outcomes in a subset of nurses who had never used prescribed sleep medication. We analyzed the association between tenure of night work and sleep outcomes (lifetime use of sleep medication, lifetime use of melatonin, nonoptimal sleep length, and SPI II score > 25) within the entire study population and within current night workers (having performed night shifts during the past 6 mo). We also analyzed the association between years since last performed night shifts and sleep outcomes among nurses who ever conducted night work. Finally, we assessed the association between night-shift frequency and sleep outcomes. We established tests for trends by estimating linear regression analyses including the OR of the logistic regression models in the model for the different night-shift work categories describing night work in years, duration since last performed night work and frequency of night work (using the LME4 function of the LMER package for R (Bates et al. 2009)). The subsets and varying definitions for sleep and night work analyses in this study are described in SupplementaryS2 Appendix Conceptual design framework.

## Results

### General characteristics

Participants who ever worked night shifts were comparable to participants who never worked night shifts for most general characteristics (Table 1). Eighty percent of participants ever worked night shifts. Of those participants, 65% provided detailed information on different aspects of their night-shift work (complete participation). Nurses who worked night shifts did this on average for 11.9 yr (SD 8.4). Night-shift workers are more often reported to be an evening type (35.8% compared to 29.9%) and slightly higher lifetime use of sleep medication (6.8% compared to 5.3%) and antidepressant medication (8.3% versus 7.8%). SupplementaryS3 Appendix presents the general characteristics stratified on recent (preceding 4 wk) performing night work. Similar patterns were observed with a more pronounced difference in melatonin use between recent night workers and day workers (9.3% versus 6.2%, respectively).

**Table 1.** General characteristics (*n* = 59,947) ever/never performed night-shift work.

	Ever performed night work (*n* = 48,081, 80%)	Never performed night work (*n* = 11,866, 20%)
Age (mean ± SD)	47.0 ± 10.8	46.4 ± 11.8
Educational level (% with applied sciences or academic degree)	47.6	46.2
Marital status (% married/cohabiting)	80.5	80.7
Birth origin (% Dutch)	96.5	96.3
BMI (mean ± SD)	24.9 ± 4.2	24.7 ± 4.1
Physical activity hours per week (mean ± SD)	3.3 ± 4.1	3.4 ± 4.4
Smoking duration in years (mean ± SD)	9.0 ± 11.7	8.6 ± 11.7
Alcohol consumption (% drinking 6 to 7 d/wk)	11.7	12.1
Diurnal preference (% more evening than morning preference and obvious evening preference)	35.8	29.9
Lifetime use of sleep medication (% ever used)	6.8	5.3
Lifetime use of melatonin (% ever used)	7.5	5.0
Antidepressant medication (% ever used)	8.3	7.8
SPI II score (mean ± SD)	20.4 ± 5.9	20.3 ± 5.8
Sleep duration in hours (mean ± SD)	7.1 ± 1.0	7.2 ± 1.0
Night work history in years	11.9 ± 8.4	–

### Associating night work with sleep outcomes

#### Ever working night shifts:

We observed an elevated OR for lifetime use of sleep medication when comparing participants ever working night shifts to participants never working night shifts after adjusting for potential confounders (OR 1.24; 95% CI 1.13–1.35, Table 2). This effect was not modified by age or diurnal preference (SupplementaryS6 Appendix). We observed similar associations between night-shift work and sleep medication (OR 1.24; 95% CI 1.14–1.36, SupplementaryS5 Appendix) among a subgroup of nurses who started using sleep medication during a period in which they also worked night shifts (74.4% of total sleep medication users). For melatonin use, the OR was elevated within participants ever working night shifts as compared to participants never working night shifts (OR 1.55; 95% CI 1.40–1.71, Table 2). The adjusted OR on nonoptimal sleep length (averaged over the preceding 4 wk) was not elevated for nurses who ever worked night shifts (OR 1.03; 95% CI 0.98–1.08, Table 2). This association was not modified by age or diurnal preference (SupplementaryS6 Appendix). Ever working night shifts did not significantly affect the OR on an SPI II score > 25 (OR 0.98; 95% CI 0.93–1.04, Table 2). This association was modified by age with a stronger effect with increasing age (OR 1.03; 95% CI 0.96–1.12 ≥ 45 yr, SupplementaryS6 Appendix) but not by diurnal preference. Within a subset of nurses who never used prescribed sleep medication (SupplementaryS7 Appendix), we observed a higher OR for lifetime use of melatonin (OR 1.54; 95% CI 1.38–1.72) compared to never having used melatonin. For nonoptimal sleep length in hours and the SPI II score, no association was observed.

**Table 2.** Logistic regression analyses associating working at night with sleep outcomes (lifetime use of sleep medication, lifetime use of melatonin, nonoptimal sleep length, and SPI II score >25 (*n* = 59,947)).

	*Ever working night shifts* *(n = 48,081) compared to never working night shifts*			*Recently working night shifts* *(n = 15,705) compared to not working night shifts in the preceding 4 wk*		
	OR	lcl, ucl 95% CI	*P*-value	OR	lcl, ucl 95% CI	*P*-value
**Lifetime use of sleep medication (yes/no)**						
*Crude*	1.27	1.16, 1.38	<0.001	1.16	1.07, 1.25	<0.001
*Adjusted*	1.24	1.13, 1.35	<0.001	1.13	1.05, 1.22	0.002
**Lifetime use of melatonin (yes/no)**						
*Crude*	1.53	1.39, 1.69	<0.001	1.73	1.61, 1.87	<0.001
*Adjusted*	1.55	1.40, 1.71	<0.001	1.72	1.59, 1.86	<0.001
**Nonoptimal sleep length (<7, ≥9 h sleep on average during the preceding 4 wk)**						
*Crude*	1.04	0.99, 1.09	0.135	1.01	0.96, 1.06	0.723
*Adjusted*	1.03	0.98, 1.08	0.265	0.98	0.94, 1.03	0.421
**SPI II score > 25**						
*Crude*	1.04	0.98, 1.10	0.195	0.99	0.94, 1.04	0.633
*Adjusted*	0.98	0.93, 1.04	0.545	0.88	0.84, 0.93	<0.001

Crude models are adjusted for age and BMI.

Adjusted models are adjusted for age, BMI, alcohol consumption, physical activity, marital status, birth origin, highest achieved level of education, diurnal preference, and smoking.

#### Recently working night shifts:

Results for recently working night shifts (preceding 4 wk) were similar to the associations between ever-working night shifts and sleep outcomes (Table 2). An elevated OR was observed when associating recently working night shifts with lifetime use of prescribed sleep medication (OR 1.13; 95% CI 1.05–1.22) and lifetime melatonin use (OR 1.72; 95% CI 1.59, 1.86). Recently working night shifts was not associated with nonoptimal sleep length averaged over the preceding 4 wk (adjusted OR 0.98; 95% CI 0.94, 1.03, Table 2). When assessing recently working night shifts, the odds of having a SPI II score > 25 were significantly lower for night workers (OR 0.88; 95% CI 0.84–0.93, Table 2) and a negative interaction with age was observed (OR 0.81; 95% CI 0.75–0.87 ≥ 45 yr, SupplementaryS6 Appendix V). Directly after working a night shift (between two consecutive night shifts), the OR on nonoptimal sleep length was significantly higher (OR 3.14; 95% CI 3.01–3.28, SupplementaryS4 Appendix).

#### Tenure of night-shift work in years:

A significant positive trend was observed for the tenure of night work in years and lifetime use of sleep medication (*P*-value for trend < 0.001, Table 3), and lifetime use of melatonin (*P*-value for trend < 0.001). We observed no significant trend for the tenure of night work in years and nonoptimal sleep length (*P*-value of 0.430 for trend). More experienced night workers showed a significantly lower OR on SPI II scores > 25 (*P*-value for trend 0.019). When we conducted these analyses on current night workers, we observed an inverse association between tenure of night-shift work and the odds of lifetime use of prescribed sleep medication (*P*-value for trend 0.036, SupplementaryS8 Appendix, Table 1). In contrast, a positive trend was observed for the tenure of night-shift work and use of melatonin, when restricting to current night workers (*P*-value for trend 0.019, SupplementaryS8 Appendix, Table 1). As for ever working night shifts, we observed increasing sleep problems (a score of > 25 on the SPI II) (*P*-value for trend 0.034) related to the tenure of night work and no significant trend for nonoptimal sleep length (*P*-value for trend 0.421).

**Table 3.** Logistic regression analyses associating tenure of night-shift work with sleep outcomes (lifetime use of sleep medication, lifetime use of melatonin, nonoptimal sleep length, and SPI II score > 25 (*n*=59,947)).

	OR	lcl, ucl 95% CI	*P*-value
**Lifetime use of sleep medication (yes/no)** Tenure of night-shift work:			
<5 yr (reference category)			
5 to 10 yr	1.12	1.02, 1.24	0.016
10 to 15 yr	1.10	0.99, 1.23	0.071
15 to 20 yr	1.17	1.03, 1.32	0.015
>20 yr	1.28	1.15, 1.42	<0.001
*Trend analysis*			*<0.001*
**Lifetime use of melatonin (yes/no)** Tenure of night-shift work:			
<5 yr			
5 to 10 yr	1.14	1.03, 1.26	0.017
10 to 15 yr	1.19	1.06, 1.33	0.003
15 to 20 yr	1.45	1.28, 1.65	<0.001
>20 yr	1.61	1.45, 1.79	<0.001
*Trend analysis*			*<0.001*
**Nonoptimal sleep length (<7, ≥9 h sleep on average during the preceding 4 wk)** Tenure of night-shift work:			
<5 yr (reference category)			
5 to 10 yr	1.02	0.96, 1.07	0.537
10 to 15 yr	1.04	0.98, 1.10	0.259
15 to 20 yr	1.05	0.98, 1.13	0.205
>20 yr	1.01	0.95, 1.08	0.702
*Trend analysis*			*0.430*
**SPI II score >25** Tenure of night-shift work:			
<5 yr (reference category)			
5 to 10 yr	0.97	0.91, 1.03	0.314
10 to 15 yr	0.93	0.87, 1.00	0.043
15 to 20 yr	0.96	0.88, 1.05	0.386
>20 yr	0.92	0.85, 0.99	0.021
*Trend analysis*			*0.019*

Models are adjusted for age, BMI, alcohol consumption, physical activity, marital status, birth origin, highest achieved level of education, diurnal preference, and smoking. We conducted trend analyses by employing linear regression models, and the italicized values represent the overall p-value for these trend analyses. <0.05 is considered statistically significant.

#### Frequency of night shifts:

We observed a positive trend for the average frequency of night shifts and lifetime use of prescribed sleep medication (*P*-value for trend < 0.001, Table 4) and melatonin use (*P*-value for trend < 0.001). We observed no clear trend for how often nurses worked night shifts and the odds of nonoptimal sleep length (*P*-value for trend 0.836). The frequency of night shifts was negatively associated with the odds of an SPI II score of > 25 (*P*-value for trend < 0.001). Within current night workers (SupplementaryS8 Appendix, Table 2), we observed an indication for a positive trend; working night shifts more often was associated with higher ORs on lifetime use of sleep medication (*P*-value for trend < 0.001). and melatonin use (*P*-value for trend < 0.001). Night workers who reported “always” working night shifts had an OR of 2.29 (95% CI 1.45–3.50) on lifetime sleep medication use. We observed no clear trend for the OR on nonoptimal sleep length and the odds on an SPI II score of > 25.

**Table 4.** Logistic regression analyses associating night-shift frequency with sleep outcomes (lifetime use of sleep medication, lifetime use of melatonin, nonoptimal sleep length, and SPI II score > 25 (*n* = 59,947)).

	OR	lcl, ucl 95% CI	*P*-value
**Lifetime use of sleep medication (yes/no)** Frequency of night shifts over preceding 4 wk:			
Never (reference category)			
Seldom	0.92	0.74, 1.13	0.403
Sometimes	1.05	0.95, 1.15	0.346
Often	1.24	1.09, 1.41	0.001
Most of the time	1.25	0.90, 1.73	0.181
Always	1.81	1.30, 2.53	<0.001
*Trend analysis*			*<0.001*
**Lifetime use of melatonin (yes/no)** Frequency of night shifts over preceding 4 wk:			
Never (reference category)			
Seldom	1.20	0.98, 1.47	0.079
Sometimes	1.59	1.46, 1.74	<0.001
Often	2.07	1.84, 2.24	<0.001
Most of the time	2.07	1.54, 2.80	<0.001
Always	1.75	1.20, 2.55	0.004
*Trend analysis*			*<0.001*
**Nonoptimal sleep length (<7, ≥9 h sleep on average during the preceding** 4 wk) Frequency of night shifts over preceding 4 wk:			
Never (reference category)			
Seldom	0.91	0.81, 1.02	0.113
Sometimes	0.93	0.88, 0.98	0.007
Often	1.09	1.00, 1.18	0.040
Most of the time	1.15	0.94, 1.41	0.178
Always	1.09	0.85, 1.39	0.488
*Trend analysis*			*0.836*
**SPI II score >25** Frequency of night shifts over preceding 4 wk:			
Never (reference category)			
Seldom	0.93	0.82, 1.06	0.253
Sometimes	0.89	0.83, 0.94	<0.001
Often	0.87	0.80, 0.96	0.003
Most of the time	0.95	0.75, 1.19	0.645
Always	0.73	0.54, 0.99	0.041
*Trend analysis*			*<0.001*

Models are adjusted for age, BMI, alcohol consumption, physical activity, marital status, birth origin, highest achieved level of education, diurnal preference and smoking. We conducted trend analyses by employing linear regression models, and the italicized values represent the overall p-value for these trend analyses. <0.05 is considered statistically significant.

#### Duration in years since last night shift (within workers that ever-performed night work):

We observed a decreasing trend for the odds-on lifetime use of prescribed sleep medication (*P*-value for trend 0.002, Table 5) and lifetime melatonin use (*P*-value for trend < 0.001, Table 5) with years since last performed night work. We did not observe a similar trend for nonoptimal sleep length (*P*-value for trend 0.979). We did observe a small yet significant effect (OR 1.11; 95% CI 1.01–1.23) on nonoptimal sleep length within a subgroup of nurses who worked night shifts 3 to 5 yr ago. Finally, we found a modest trend for the years since last performed night work and the odds on a SPI II score of > 25 (*P*-value for trend 0.039). The categories of nurses who performed night work 1 to 3 yr ago, 3 to 5 yr ago, and >20 yr ago were significantly associated with increased ORs on SPI II scores > 25 (respectively OR 1.15; 95% CI 1.03–1.28, OR 1.15; 95% CI 1.03–1.29, and OR 1.11; 95% CI 1.02–1.21, Table 5).

**Table 5.** Logistic regression analyses associating duration in years since last performed night shifts with sleep outcomes (lifetime use of sleep medication, lifetime use of melatonin, nonoptimal sleep length, and SPI II score > 25, within participants ever working nights (*n* = 48,081)).

	OR	lcl, ucl 95% CI	*P*-value
**Lifetime use of sleep medication (yes/no)** Duration in years since last performed night work:			
<1 yr (reference category)			
1 to 3 yr	1.13	0.96, 1.32	0.130
3 to 5 yr	1.02	0.86, 1.21	0.800
5 to 10 yr	1.00	0.87, 1.15	0.990
10 to 20 yr	0.97	0.87, 1.10	0.660
>20 yr	0.83	0.74, 0.94	0.002
*Trend analysis*			*0.002*
**Lifetime use of melatonin (yes/no)** Duration in years since last performed night work:			
<1 yr (reference category)			
1 to 3 yr	0.92	0.79, 1.07	0.294
3 to 5 yr	0.84	0.71, 0.99	0.046
5 to 10 yr	0.77	0.67, 0.88	<0.001
10 to 20 yr	0.63	0.56, 0.71	<0.001
>20 yr	0.61	0.54, 0.69	<0.001
*Trend analysis*			*<0.001*
**Nonoptimal sleep length (<7, ≥9 h sleep on average during the preceding 4 wk)** Duration in years since last performed night work:			
<1 yr (reference category)			
1 to 3 yr	1.01	0.92, 1.11	0.807
3 to 5 yr	1.11	1.01, 1.23	0.039
5 to 10 yr	1.03	0.95, 1.11	0.536
10 to 20 yr	1.02	0.95, 1.09	0.684
>20 yr	1.00	0.94, 1.08	0.921
*Trend analysis*			*0.979*
**SPI II score >25** Duration in years since last performed night work:			
<1 yr (reference category)			
1 to 3 yr	1.15	1.03, 1.28	0.010
3 to 5 yr	1.15	1.03, 1.29	0.016
5 to 10 yr	1.08	0.98, 1.19	0.125
10 to 20 yr	1.08	1.00, 1.17	0.066
>20 yr	1.11	1.02, 1.21	0.013
*Trend analysis*			*0.039*

Models are adjusted for age, BMI, alcohol consumption, physical activity, marital status, birth origin, highest achieved level of education, diurnal preference, and smoking. We conducted trend analyses by employing linear regression models, and the italicized values represent the overall p-value for these trend analyses. <0.05 is considered statistically significant.

## Discussion

In a large study of approximately 60,000 Dutch nurses, we observed that ever working at night and both tenure of night-shift works and average frequency of night shifts was related to increased lifetime use of prescribed sleep medication and melatonin but not to other metrics of sleep quality such as nonoptimal sleep length and self-reported sleep quality. The association between lifetime medication and melatonin use weakened with years since the last night shift.

Our results extend the observation of reported acute sleep problems while performing night-shift work (Gold et al. 1992; Arendt et al. 2006; Grundy et al. 2009) to sleep problems due to lifetime long-term night-shift work. Our observation of nonoptimal sleep length directly after working a night shift was in line with previous studies also reporting large differences when assessing acute effects (Gold et al. 1992; Arendt et al. 2006; Grundy et al. 2009), and also with studies measuring sleep using actimetry (Axelsson et al. 2004; Niu et al. 2017). Our observation of increased lifetime use of prescribed sleep medication and melatonin associated with night-shift work is also in line with previous studies. Waage et al. in a longitudinal study among Norwegian nurses also reported an increased use of melatonin (Waage et al. 2014). One large cohort study of Finnish public sector employees previously linked night-shift work with sleep medication (Tucker et al. 2021). Age-stratified Cox proportional hazard regression models were computed to examine the use of medication comparing night-shift workers with day workers, with up to 11 yr of follow-up (Tucker et al. 2021). Night-shift work was associated with increased use of sleep medication in all age groups, suggesting that circadian disruption puts night-shift workers at increased risk of developing clinically significant levels of sleep problems (Tucker et al. 2021). Nevertheless, this previously conducted cohort study lacked detailed information regarding the frequency of night shifts and diurnal preference, thereby limiting the interpretation of the findings (Tucker et al. 2021).

Interpretation of our results is challenging due to the observed association with medication use but not with reported sleep problems using (self-reported) established sleep quality metrics. Additionally, a significant upward trend was found between the tenure and frequency of night-shift work and the use of sleep medication, with an opposite trend for sleep medication, not melatonin use, when we restricted the analysis to current night workers. Note however that in these analyses the reference category is different. Where in the analyses of lifetime night-shift work the reference category includes subjects that never performed night-shift work the reference in the current night-shift work analyses are nurses performing night shifts for a shorter period. These results suggest possible selection mechanisms, where recent night-shift workers show higher sleep medication use, which diminishes over time as nurses with a dependency on sleep medication might leave the profession earlier.

Our interpretation of the result hinges on the assumption that increased use of prescribed and over-the-counter (OTC) medication is indicative of sleep problems. As we do not observe a clear association with reported sleep problems in this population, this might suggest that the used medication is effective in combating sleep problems. In addition, the outcome of “non-optimal sleep” was more affected by measurement error than sleep medication. Even though this information was collected retrospectively, in conjunction with the questions on periods working night shifts, it is essential to consider the implications of using sleep medication as an outcome measure. Sleep medication, unlike self-reported sleep quality, leaves less room for individual interpretation, as participants need to establish factual usage, thereby limiting the chance of errors and recall bias. The effects of night work on the use of melatonin may be more direct, as melatonin is readily available and accessible OTC, in contrast to medication prescribed by general practitioners (Lawman et al. 2016). Also, as melatonin is an OTC medication the seriousness of using melatonin versus prescribed medication might be perceived differently with less nurses leaving the profession or an indication of less severe sleep problems that lead to less nurses leaving the profession because of these problems. Still, we must acknowledge the potential for measurement error in assessing sleep medication use. Nevertheless, using medication as an outcome measure enables us to detect effects that would not have been possible if we solely relied on self-reported sleep quality. In the subset of nurses who never took sleep medication, we observed a borderline significant OR on nonoptimal sleep length (<7, ≥9 h sleep on average during the preceding 4 wk) for nurses ever working night shifts. The SPI II score was still higher for nurses recently working nights in this subset. It is important to note that in this subset since we removed the nurses ever using prescribed sleep medication, we also potentially removed the nurses who are most susceptible to developing sleep problems as a result of working night shifts. When analyzing sleep represented by a recall period of 4 wk, we observed only small differences in sleep length after night work. It is possible that shift workers might perceive their sleep-related problems directly after a night shift as an exception, and therefore not consider their sleep to be disturbed on average (over the preceding 4 wk), even though their use of sleep medication does indicate more sleep-related problems. Akerstedt et al. indicated that it might be difficult for a participant to give a weighted answer about their overall sleep performance and that shift workers might consider sleep disturbance “part of the job” (Akerstedt et al. 2008).

Our analyses suggested reversibility of the observed effects with clear reductions of the magnitude of the association after time since last performing night shifts. We saw the strongest negative effects on perceived sleep problems when participants had performed night work 1 to 3 and 3 to 5 yr ago. Moreover, there was a downward trend for the odds-on lifetime sleep medication use and time since last performed night shifts. Härmä et al*.* (2018) demonstrated that when participants changed their schedule from shift work to day work, feeling of fatigue significantly decreased, although this might take several years. Niedhammer et al. (1994) also described reversibility, sleep disorders decreased significantly after leaving shift work (Niedhammer et al. 1994).

Diurnal preference did not modify the association between recently working night shifts and sleep-problem outcomes that is not in line with the literature. A study by Reinke et al. (2015) reflected clear differences between diurnal preferences in sleeping before and after a night shift (Reinke et al. 2015). Furthermore, in a review by Saksvik et al. on factors which might explain tolerance towards night work, most studies supported that “evening types” are associated with high tolerance towards night work and being a “morning type” resulted in shorter sleep durations and higher levels of sleep disturbance in response to night-shift work (Saksvik et al. 2011), although results varied. Since we defined less than 7 h or 9 h or more as nonoptimal sleep length (Viala-Danten et al. 2008a), we cannot distinguish the differences between long and short sleep lengths for diurnal preferences, it is conceivable that morning types resulted in shorter sleep durations (or vice versa).

Studying night work in relation to sleep quality is important due to the significant impact of chronic sleep deprivation on overall health. Chronic lack of sleep quality has been demonstrated to have strong associations with various chronic diseases, including breast cancer risk (Wang et al. 2015), cardio-metabolic disorders and all-cause mortality (Pinheiro et al. 2006; Hublin et al. 2007; Kline et al. 2012; Ford et al. 2014; Wang et al. 2014), and are therefore a plausible pathway between night-shift work and severe health issues. By focusing on this outcome, our research aims to shed light on the potential consequences of night work on sleep quality, enabling us to better understand the risks and implications for shift workers’ health. The findings from our study hold valuable insights that can help guide targeted interventions. For instance, our results may inform consultations regarding the use of sleep medications among shift workers.

Our study has some limitations and strengths. The response rate of this study was 31% that could limit the external validity/generalizability of our results. The study population of this large national cohort is representative of the total population of Dutch female nurses with regard to age and educational level (Pijpe et al. 2014). Since our study sample included only female healthcare workers, caution is needed when extrapolating our findings to other industries and sectors that employ night-shift workers and male workers. A relatively small percentage of the nurses in our study used sleep medication (approximately 6%). Due to the sheer size of our study population, we had sufficient statistical power to assess associations. While statistically significant results can be obtained with a large cohort, we acknowledge the possibility of small effect sizes that may not be clinically or practically meaningful, therefore we primarily relied on trend tests to examine associations between shift work and sleep outcomes. Due to the retrospective character of the detailed questions regarding job histories and shift work schedules, which is rare for large cohort studies, we were able to distinguish different aspects of night-shift work such as tenure (duration in years) and the number of years since participants last performed night shifts. Sleep was measured using the MOS Sleep scale, the reliability and validity of this often-used scale have been demonstrated in several studies (Smith and Wegener 2003; Allen et al. 2009). Our use of self-reported data may introduce errors in both exposure and outcome information, which could be nondifferential or potentially differential if memory errors are more likely in night workers due to sleep-related problems. Due to the cross-sectional nature of our analyses, we should consider potential attrition bias (selective drop-out) in interpreting our findings on sleep outcomes. Specifically, individuals who previously worked night shifts and experienced health complaints including sleep disturbances may have left the profession, and consequently lost their registration, and as a result may not have been invited into the Nightingale cohort. The healthy-worker effect therefore may have influenced our results, possibly attenuating associations and may have contributed to the absence of strong associations between night shift and sleep quality metrics in our study. The healthy-worker effect therefore may have influenced our results, possibly attenuating associations and more likely so for sleep disturbances (past 4 wk) than for sleep medication use (lifetime). This may have contributed to the absence of strong associations between night-shift work and sleep disturbance in our study. Our finding of increased sleep medication use in night workers may nonetheless point to undetected sleep problems, perhaps in other sleep domains than assessed in our study (e.g. sleep regularity) and merits investigation. The observed association between night-shift work and sleep disturbances and lifetime sleep medication usage provide valuable insights into the long-term effects of night-shift work on sleep quality. Our data relies on self-reported information, as schedules of shift work conducted by participants over many years and locations were not archived by employers. Participants were asked to recall their lifetime shift work exposure at an average age of 46.9 yrs, which may introduce nondifferential misclassification. About one-third of participants who reported shift work did not complete the detailed section, possibly due to questionnaire fatigue or recall complexity. However, we derived the type and tenure of night-shift work from the occupational history section and we utilized multiple imputations to address missing data. More information regarding the strengths and weaknesses of the Nightingale study design is described by Pijpe et al. (2014).

Further research is needed to investigate the potential long-term health consequences of sleep problems related to night-shift work. Examining the association between night-shift work, sleep disturbances, and various health outcomes like cardiovascular disease, metabolic disorders, and cancer would enhance our understanding of the overall health risks associated with this type of work schedule. Longitudinal studies following individuals over time would offer valuable insights into the chronic effects of night-shift work on sleep quality, allowing a comprehensive understanding of its long-term impact and the potential reversibility of sleep problems after leaving night-shift work. The Nightingale study presents an opportunity for a longitudinal follow-up study. Exploring individual differences, such as chronotype (morningness/eveningness preference) and genetic factors, in the susceptibility to sleep problems caused by night-shift work would provide insights into the interplay between circadian rhythms and sleep disturbances. Additionally, it is crucial to consider other factors affecting females’ sleep, including hormonal fluctuations during the menstrual cycle (Baker et al. 2007) and during the menopausal transition and the stress related to managing multiple roles, such as work and family responsibilities, which may contribute to sleep disturbances among female shift workers (Chung et al., 2009).

## Conclusion

We described associations between lifetime night-shift work and lifetime use of prescribed and OTC medication use indicative of long-term sleep problems. Given the importance of compromised sleep quality in developing chronic health problems, results from our study suggest further research into the role of sleep quality in studies of shift work and disease is warranted. The indication of reversibility of the effects is important but demonstrates on the other hand the potential long lingering effect of shift work on sleep.

## Supplementary Material

wxad058_suppl_Supplementary_Appendix

## Data Availability

The data underlying this article will be shared on reasonable request to the corresponding author.
