# Supplementary material for: Associating night-shift work with lifetime use of sleep medication and sleep quality in a cohort of female nurses
Source: Ann Work Expo Health. 2023 Sep 27;67(9):1056–68. doi: 10.1093/annweh/wxad058 (PMC10752347; doi:10.1093/annweh/wxad058)
Supplement: wxad058_suppl_Supplementary_Appendix [file wxad058_suppl_supplementary_appendix.docx]

Associating night-shift work with lifetime use of sleep medication and sleep quality in a cohort of female nurses

*Daniëlla van de Langenberg^a^ MSc, Jelle Vlaanderen^a^ PhD*, Nina Berentzen^b^ PhD, Hans Kromhout^a^ PhD, and Roel Vermeulen^a^ PhD*

*Corresponding author

IRAS, Institute for Risk Assessment Sciences, Utrecht University

Yalelaan 2, 3508 TD Utrecht, the Netherlands

T: +31 30253 89 50 / E: [J.J.Vlaanderen@uu.nl](mailto:J.J.Vlaanderen@uu.nl)

Affiliations:

1. IRAS, Institute for Risk Assessment Sciences, Utrecht University, the Netherlands
2. NKI, Netherlands Cancer Institute, Amsterdam, the Netherlands

**S1 Appendix MOS dimensions and construction of SPI II**

Table 1

| MOS dimensions and SPI II | **Item #** | **Item content** | **Answer possibilities** |
| --- | --- | --- | --- |
| Quantity of sleep / non-optimal sleep | 2 | How many hours did you sleep on average during the past 4 weeks? | In rounded hours, 7 or 8 hours is considered optimal, all other answers were considered non-optimal (<7, ≥9 hours sleep) |
| Sleep disturbance | 1 | How long did it usually take to fall asleep during the past 4 weeks? | 0-15 minutes, 16-30 minutes, 31-45 minutes, 46-60 minutes, >60minutes |
|  |  | **How often during the past 4 weeks did you:** |  |
|  | 3 | … feel that your sleep was not quiet (moving restlessly, feeling tense)? | All of the time, most of the time, a good portion of the time, some of the time, a little of the time, none of the time |
|  | 7 | … have trouble falling asleep? | ,, |
|  | 8 | … awaken during your sleep time and have trouble falling asleep again? | ,, |
| Somnolence | 6 | … feel drowsy or sleepy during the day? | ,, |
|  | 9 | … have trouble staying awake during the day? | ,, |
|  | 11 | … take naps during the day (>5 minutes)? | ,, |
| Sleep adequacy | 4 | … get enough sleep to feel rested upon waking? | ,, |
|  | 12 | … get the amount of sleep needed? | ,, |
| Snoring | 10 | … snore during your sleep? | ,, |
| Respiratory | 5 | … awaken short of breath or with a headache? | ,, |
| SPI II | 1, 3:9, 12 | All items except for 4 and 12 are reversed scored, 0-100 scale |  |

**S2 Appendix Conceptual design framework: subsets and varying definitions for sleep and night work analyses in this study**


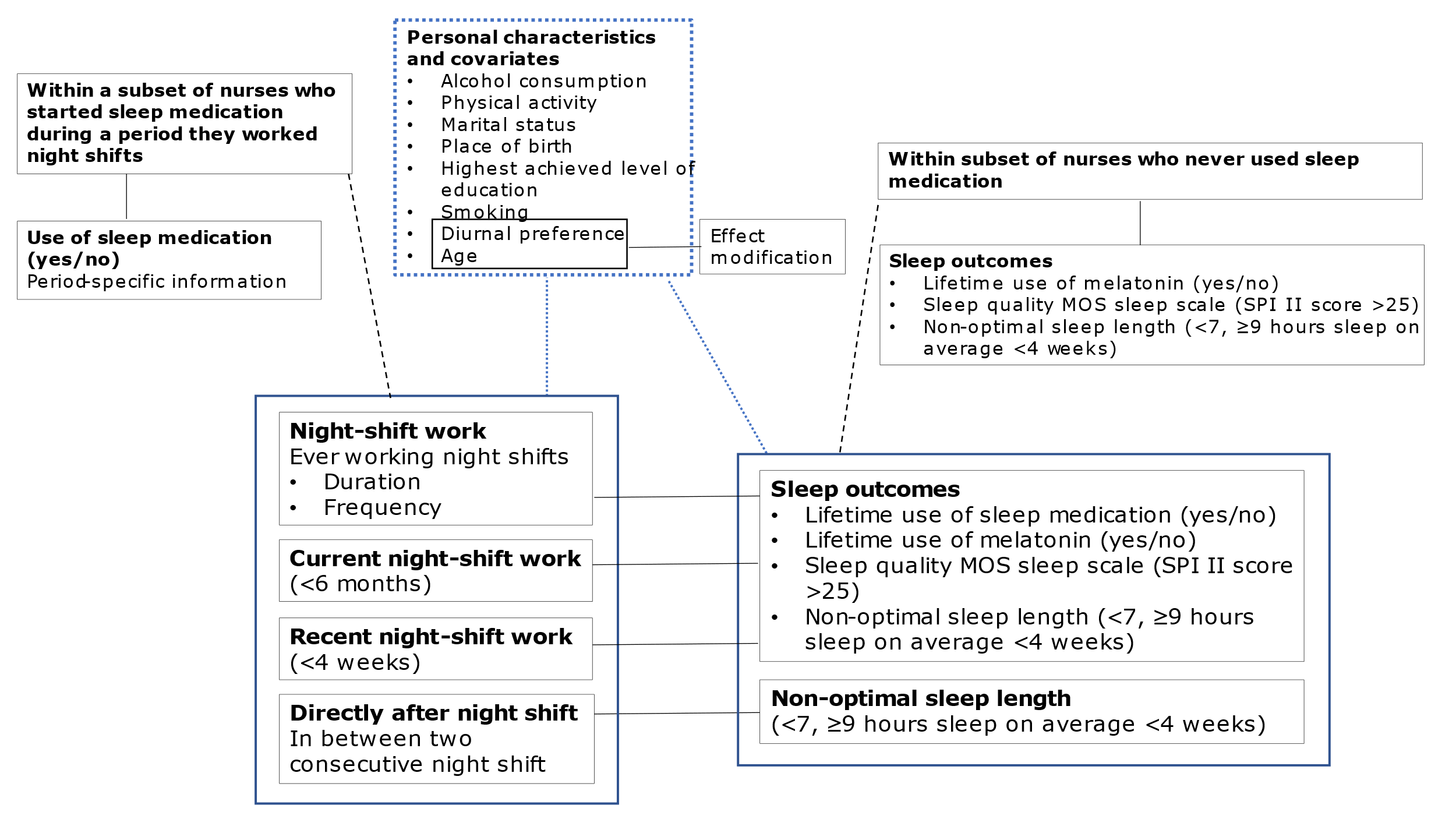


Figure 1. Conceptual design framework: subsets and varying definitions for sleep and night work analyses in this study

**S3 Appendix General characteristics table stratifying night-shift work on the preceding four weeks (recently working night shifts)**

Table 1: General characteristics (n=59,947)

Night work is defined as recently working night shifts (having performed night work the preceding four weeks)

|  | Night workers preceding four weeks (n=15,705, 26%) | Day workers preceding four weeks (n=44,242, 74%) |
| --- | --- | --- |
| Age (mean ± SD) | 43.0 ± 11.5 | 48.3 ± 10.5 |
| Educational level (% with applied sciences or academic degree) | 41 | 49.5 |
| Marital status (% married / cohabiting) | 76.5 | 82.1 |
| Birth origin (% Dutch) | 96.2 | 96.5 |
| Body mass index (mean ± SD) | 24.8 ± 4.3 | 24.9 ± 4.1 |
| Physical activity hours per week (mean ± SD) | 3.3 ± 4.1 | 3.4 ± 4.2 |
| Smoking duration in years (mean ± SD) | 7.8 ± 11.1 | 9.3 ± 11.9 |
| Alcohol consumption (% drinking 6-7 days a week) | 7.5 | 13.3 |
| Diurnal preference (% more evening than morning preference, and obvious evening preference) | 42.8 | 31.7 |
| Lifetime use of sleep medication (% ever used) | 6.9 | 6.5 |
| Lifetime use of melatonin (% ever used) | 9.3 | 6.2 |
| Antidepressant medication (% ever used) | 5.8 | 9.0 |
| SPI II score (mean ± SD) | 20.7 ± 5.7 | 20.3 ± 5.9 |
| Sleep duration in hours (mean ± SD) | 7.2 ± 0.9 | 7.1 ± 1 |
| Night work history in years | 13.4 ± 10.3 | 8.6 ± 8.8 |

**S4 Appendix Logistic regression analyses for non-optimal sleep length directly after a night shift, in between two consecutive night shifts**

Table 1: Logistic regression analyses for non-optimal sleep length directly after a night shift (between two consecutive night shifts) compared to sleep on average over the preceding four weeks (n=14,817)

|  | Odds ratio | lcl, ucl 95% CI | *p*-value |
| --- | --- | --- | --- |
| Non-optimal sleep length (<7, ≥9 hours sleep) |  |  |  |
| *Crude* | 3.11 | 2.98, 3.24 | <0.001 |
| *Adjusted* | 3.14 | 3.01, 3.28 | <0.001 |

*Crude models are adjusted for age and BMI*

*Adjusted models are adjusted for age, BMI, alcohol consumption, physical activity, marital status, birth origin, highest achieved level of education, diurnal preference and smoking*

**S5 Appendix Logistic regression analyses within nurses who started using sleep medication during a time period in which they worked night shifts**

Table 1: Logistic regression analyses associating working at night with sleep medication use, within nurses who started their sleep medication during a period in which they worked night shifts (n=58,903), reference category is never having used sleep medication during lifetime

|  | *Ever working night shifts compared to never working night shifts* | | | *Recently working night shifts compared to not working night shifts in the preceding four weeks* | | |
| --- | --- | --- | --- | --- | --- | --- |
|  | Odds ratio | lcl, ucl 95% CI | *p*-value | Odds ratio | lcl, ucl 95% CI | *p*-value |
| **Use of sleep medication (yes/no)** |  |  |  |  |  |  |
| *Crude* | 1.27 | 1.16, 1.38 | <0.001 | 1.15 | 1.07, 1.24 | <0.001 |
| *Adjusted* | 1.24 | 1.14, 1.36 | <0.001 | 1.10 | 1.02, 1.19 | 0.019 |

*Crude models are adjusted for age and BMI*

*Adjusted models are adjusted for age, BMI, alcohol consumption, physical activity, marital status, birth origin, highest achieved level of education, diurnal preference, and smoking*

**S6 Appendix** **Effect modification diurnal preference and age**

Table 1: Logistic regression analyses analyzing effect modification for age and diurnal preference for the association between working at night and sleep outcomes (lifetime use of sleep medication, non-optimal sleep length, and SPI II score >25) (n=59,947)

|  | *Ever working night shifts compared to never working night shifts* | | | *Recently working night shifts compared to not working night shifts in the preceding four weeks* | | |
| --- | --- | --- | --- | --- | --- | --- |
|  | Odds ratio | lcl, ucl 95% CI | *p*-value | Odds ratio | lcl, ucl 95% CI | *p*-value |
| **Lifetime use of sleep medication (yes/no)** |  |  |  |  |  |  |
| *Age * night work* | -0.01 | -0.01, 0.00 | 0.172 | -0.01 | -0.01, 0.00 | 0.183 |
| *Evening preference * night work* | 0.06 | -0.15, 0.27 | 0.573 | -0.17 | -0.35, 0.01 | 0.061 |
| *No specific preference * night work* | -0.02 | -0.24, 0.20 | 0.876 | -0.13 | -0.33, 0.07 | 0.189 |
| **Non-optimal sleep length (<7, ≥9 hours sleep on average during the preceding four weeks)** |  |  |  |  |  |  |
| *Age * night work* | 0.00 | -0.01, 0.00 | 0.317 | 0.00 | -0.01, 0.00 | 0.572 |
| *Evening preference * night work* | 0.08 | -0.04, 0.20 | 0.175 | 0.08 | -0.02, 0.19 | 0.124 |
| *No specific preference * night work* | 0.05 | -0.07, 0.17 | 0.407 | 0.05 | -0.07, 0.17 | 0.430 |
| **SPI II score >25** |  |  |  |  |  |  |
| *Age * night work* | 0.01 | 0.00, 0.01 | 0.005 | -0.01 | -0.02, -0.01 | <0.001 |
| *Evening preference * night work* | 0.09 | -0.05, 0.23 | 0.196 | 0.02 | -0.11, 0.14 | 0.812 |
| *No specific preference * night work* | -0.01 | -0.16, 0.14 | 0.913 | -0.07 | -0.21, 0.08 | 0.356 |

*Models are adjusted for age, BMI, alcohol consumption, physical activity, marital status, birth origin, highest achieved level of education, diurnal preference and smoking*

*Diurnal preference reference category = morning preference*

Table 2: Logistic regression analyses analyzing the interaction effect of age for the association between working at night and SPI II score >25 (n=59,947)

|  | *Ever working night shifts compared to never working night shifts* | | | *Recently working night shifts compared to not working night shifts in the preceding four weeks* | | |
| --- | --- | --- | --- | --- | --- | --- |
|  | Odds ratio | lcl, ucl 95% CI | *p*-value | Odds ratio | lcl, ucl 95% CI | *p*-value |
| **SPI II score >25** |  |  |  |  |  |  |
| *Age <45 years (n= 22,440)* | 0.96 | 0.88, 1.04 | 0.302 | 0.91 | 0.84, 0.98 | 0.012 |
| *Age ≥ 45 years (n= 37,507)* | 1.03 | 0.96, 1.12 | 0.395 | 0.81 | 0.75, 0.87 | <0.001 |

*Models are adjusted for age, BMI, alcohol consumption, physical activity, marital status, birth origin, highest achieved level of education, diurnal preference and smoking*

**S7 Appendix Associating working at night with sleep outcomes within a subset of nurses whom never used sleep medication**

Table 1: Logistic regression analyses associating working at night with sleep outcomes (lifetime use of sleep medication, lifetime use of melatonin, non-optimal sleep length, and SPI II score >25) within a subset of nurses whom never used sleep medication (n=56,070)

|  | ***Ever working night shifts*** | | | ***Recently working night shifts*** | | |
| --- | --- | --- | --- | --- | --- | --- |
|  | Odds ratio | lcl, ucl 95% CI | *p*-value | Odds ratio | lcl, ucl 95% CI | *p*-value |
| **Lifetime use of melatonin (yes/no)** |  |  |  |  |  |  |
| *Crude* | 1.54 | 1.38, 1.72 | <0.001 | 1.81 | 1.67, 1.97 | <0.001 |
| *Adjusted* | 1.54 | 1.38, 1.72 | <0.001 | 1.81 | 1.66, 1.97 | <0.001 |
| **Non-optimal sleep length (<7, ≥9 hours sleep on average during the preceding four weeks)** |  |  |  |  |  |  |
| *Crude* | 1.05 | 1.00, 1.11 | 0.051 | 1.01 | 0.96, 1.06 | 0.764 |
| *Adjusted* | 1.04 | 0.99, 1.10 | 0.116 | 0.98 | 0.94, 1.03 | 0.483 |
| **SPI II score >25** |  |  |  |  |  |  |
| *Crude* | 1.03 | 0.97, 1.09 | 0.355 | 0.99 | 0.94, 1.04 | 0.664 |
| *Adjusted* | 0.96 | 0.92, 1.04 | 0.415 | 0.89 | 0.84, 0.94 | <0.001 |

*Crude models are adjusted for age and BMI*

*Adjusted models are adjusted for age, BMI, alcohol consumption, physical activity, marital status, birth origin, highest achieved level of education, diurnal preference and smoking*

**S8 Appendix** **Associating tenure of night work and night-shift frequency to sleep outcomes within current night workers**

Table 1: Logistic regression analyses associating tenure of night work with sleep outcomes (lifetime use of sleep medication, lifetime use of melatonin, non-optimal sleep length, and SPI II score >25 (n=14,817))

|  | Odds ratio | lcl, ucl 95% CI | *p*-value |
| --- | --- | --- | --- |
| **Lifetime use of sleep medication (yes/no)**  Tenure of night work: |  |  |  |
| <5 years (reference category) |  |  |  |
| 5 – 10 years | 1.00 | 0.74, 1.37 | 0.998 |
| 10 – 15 years | 0.91 | 0.66, 0.26 | 0.572 |
| 15 – 20 years | 0.84 | 0.59, 1.19 | 0.323 |
| > 20 years | 0.77 | 0.56, 1.07 | 0.115 |
| *Trend analysis* |  |  | *0.036* |
| **Lifetime use of melatonin (yes/no)**  Tenure of night work: |  |  |  |
| <5 years (reference category) |  |  |  |
| 5 – 10 years | 1.14 | 0.85, 1.55 | 0.391 |
| 10 – 15 years | 1.12 | 0.82, 1.54 | 0.479 |
| 15 – 20 years | 1.52 | 1.10, 2.11 | 0.013 |
| > 20 years | 1.38 | 1.01, 1.92 | 0.050 |
| *Trend analysis* |  |  | *0.019* |
| **Non-optimal sleep length (<7, ≥9 hours sleep on average during the preceding four weeks)**  Tenure of night work: |  |  |  |
| <5 years (reference category) |  |  |  |
| 5 – 10 years | 1.28 | 1.07, 1.53 | 0.007 |
| 10 – 15 years | 1.05 | 0.87, 1.27 | 0.602 |
| 15 – 20 years | 1.16 | 0.95, 1.43 | 0.142 |
| > 20 years | 1.04 | 0.86, 0.27 | 0.688 |
| *Trend analysis* |  |  | *0.421* |
| **SPI II score >25**  Tenure of night work: |  |  |  |
| <5 years (reference category) |  |  |  |
| 5 – 10 years | 0.97 | 0.81, 1.16 | 0.743 |
| 10 – 15 years | 0.86 | 0.74, 1.05 | 0.132 |
| 15 – 20 years | 0.88 | 0.88, 0.71 | 0.271 |
| > 20 years | 0.81 | 0.81, 0.65 | 0.048 |
| *Trend analysis* |  |  | *0.034* |

*Models are adjusted for age, BMI, alcohol consumption, physical activity, marital status, birth origin, highest achieved level of education, diurnal preference, and smoking*

Table 2: Logistic regression analyses associating night-shift frequency with sleep outcomes (lifetime use of sleep medication, lifetime use of melatonin, non-optimal sleep length, and SPI II score >25 (n=14,817))

|  | Odds ratio | lcl, ucl 95% CI | *p*-value |
| --- | --- | --- | --- |
| **Lifetime use of sleep medication (yes/no)**  Frequency of night shifts over preceding 4 weeks: |  |  |  |
| Never (reference category) |  |  |  |
| Seldom | 1.17 | 0.83, 1.62 | 0.344 |
| Sometimes | 1.23 | 1.03, 1.48 | 0.027 |
| Often | 1.39 | 1.13, 1.72 | 0.002 |
| Most of the time | 1.29 | 0.82, 1.96 | 0.254 |
| Always | 2.29 | 1.45, 3.50 | <0.001 |
| *Trend analysis* |  |  | *<0.001* |
| **Lifetime use of melatonin (yes/no)**  Frequency of night shifts over preceding 4 weeks: |  |  |  |
| Never (reference category) |  |  |  |
| Seldom | 0.92 | 0.66, 1.27 | 0.625 |
| Sometimes | 1.27 | 1.07, 1.50 | 0.006 |
| Often | 1.47 | 1.21, 1.79 | <0.001 |
| Most of the time | 1.59 | 1.07, 2.30 | 0.017 |
| Always | 1.42 | 0.85, 2.27 | 0.161 |
| *Trend analysis* |  |  | *<0.001* |
| **Non-optimal sleep length (<7, ≥9 hours sleep on average during the preceding four weeks)**  Frequency of night shifts over preceding 4 weeks: |  |  |  |
| Never (reference category) |  |  |  |
| Seldom | 0.84 | 0.70, 1.01 | 0.071 |
| Sometimes | 0.77 | 0.70, 0.85 | <0.001 |
| Often | 0.93 | 0.82, 1.04 | 0.203 |
| Most of the time | 0.85 | 0.65, 1.10 | 0.226 |
| Always | 1.14 | 0.83, 1.55 | 0.409 |
| *Trend analysis* |  |  | *0.049* |
| **SPI II score >25**  Frequency of night shifts over preceding 4 weeks: |  |  |  |
| Never (reference category) |  |  |  |
| Seldom | 0.93 | 0.75, 1.14 | 0.496 |
| Sometimes | 0.91 | 0.81, 1.01 | 0.082 |
| Often | 0.93 | 0.81, 1.06 | 0.266 |
| Most of the time | 0.90 | 0.66, 1.20 | 0.463 |
| Always | 0.87 | 0.58, 1.25 | 0.459 |
| *Trend analysis* |  |  | *0.123* |

*Models are adjusted for age, BMI, alcohol consumption, physical activity, marital status, birth origin, highest achieved level of education, diurnal preference and smoking*


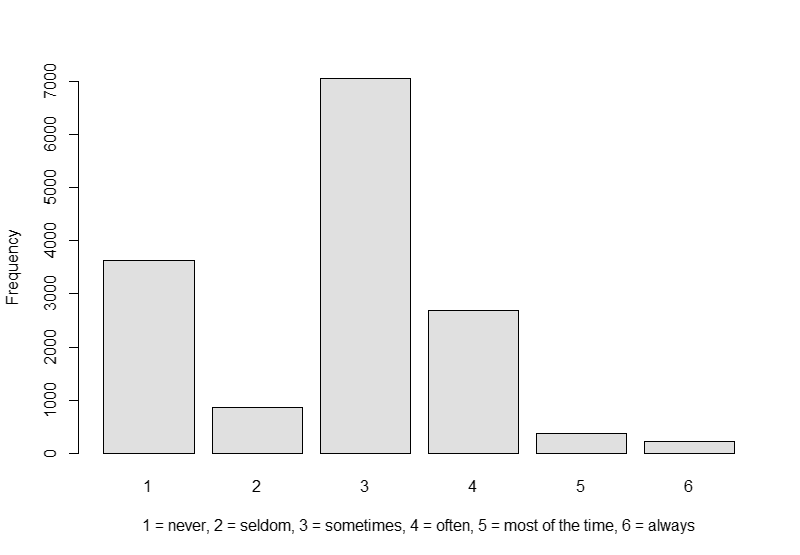


Figure 1. Frequency of night shifts over the preceding four weeks, within current night-shift workers
